# Expression of *Arabidopsis SHN1* in Indian Mulberry (*Morus indica* L.) Increases Leaf Surface Wax Content and Reduces Post-harvest Water Loss

**DOI:** 10.3389/fpls.2017.00418

**Published:** 2017-04-04

**Authors:** R. S. Sajeevan, Karaba N. Nataraja, K. S. Shivashankara, N. Pallavi, D. S. Gurumurthy, M. B. Shivanna

**Affiliations:** ^1^Department of Crop Physiology, University of Agricultural SciencesBangalore, India; ^2^Department of Studies in Applied BotanyKuvempu University, Shimoga India; ^3^Division of Plant Physiology and Biochemistry, Indian Institute of Horticultural ResearchBangalore, India; ^4^ITC Life Sciences and Technology Centre, ITC LimitedBangalore, India

**Keywords:** mulberry, *AtSHN1*, transgenic plants, epicuticular wax, post-harvest water loss, moisture retention capacity

## Abstract

Mulberry (*Morus* species) leaf is the sole food for monophagous silkworms, *Bombyx mori* L. Abiotic stresses such as drought, salinity, and high temperature, significantly decrease mulberry productivity and post-harvest water loss from leaves influence silkworm growth and cocoon yield. Leaf surface properties regulate direct water loss through the cuticular layer. Leaf surface waxes, contribute for cuticular resistance and protect mesophyll cells from desiccation. In this study we attempted to overexpress *AtSHN1*, a transcription factor associated with epicuticular wax biosynthesis to increase leaf surface wax load in mulberry. *Agrobacterium* mediated *in vitro* transformation was carried out using hypocotyl and cotyledonary explants of Indian mulberry (cv. M5). Mulberry transgenic plants expressing *AtSHN1* displayed dark green shiny appearance with increased leaf surface wax content. Scanning electron microscopy (SEM) and gas chromatograph–mass spectrometry (GC-MS) analysis showed change in pattern of surface wax deposition and significant change in wax composition in *AtSHN1* overexpressors. Increased wax content altered leaf surface properties as there was significant difference in water droplet contact angle and diameter between transgenic and wild type plants. The transgenic plants showed significant improvement in leaf moisture retention capacity even 5 h after harvest and there was slow degradation of total buffer soluble protein in detached leaves compared to wild type. Silkworm bioassay did not indicate any undesirable effects on larval growth and cocoon yield. This study demonstrated that expression of *AtSHN1*, can increase surface wax load and reduce the post-harvest water loss in mulberry.

## Introduction

Mulberry, a member of the family *Moraceae* is commercially cultivated as the sole source of food for monophagous silkworm, *Bombyx mori* L. Increase in mulberry productivity is vital for sericulture industry. India is the second largest producer of raw silk, next to Republic of China Central Silk Board, (2014–2015). To meet the silk demand, there is a need to increase mulberry leaf production. Major mulberry growing areas in India are under dry and irrigated conditions. Under these conditions, abiotic stresses such as drought, salinity, and alkalinity cause 50–60% yield loss (Rao, 2002). In addition to these constraints, post-harvest water loss due to the time lag between leaf harvest and silkworm feeding influences silkworm growth and cocoon yield (Mamrutha et al., 2010). Slow water loss from harvested leaves is a desirable trait, which helps in maintaining post-harvest leaf quality. Since stomata close soon after the leaves are detached, leaf surface waxes play a significant role in regulating moisture loss. The importance of leaf surface wax in regulating leaf moisture loss has been demonstrated and a positive correlation between surface wax load and moisture retention capacity (MRC) has been shown in mulberry (Mamrutha et al., 2010).

Leaf surface waxes are complex mixture of very-long-chain fatty acids and their derivatives, produced through complex biochemical pathways (Jenks et al., 1996, 2002; Samuels et al., 2008; Kunst and Samuels, 2009). Chemical characteristics of the cuticular wax, surface wax load, and wax crystal morphology are the primary determinants of the permeability of the plant cuticle (Schreiber et al., 1996; Mamrutha et al., 2010, 2017). Considerable progress has been made in understanding the genetic determinants of the biosynthesis of cutin and cuticular waxes in model plants (Kunst et al., 2006; Kunst and Samuels, 2009; Yeats and Rose, 2013; Lee and Suh, 2015a). A number of upstream regulatory proteins such as transcriptions factors (TFs) that coordinate the expression of downstream target genes associated with wax biosynthesis have been shown to alter leaf surface wax load (Broun et al., 2004; Seo et al., 2011; Borisjuk et al., 2014; Park et al., 2016). Many regulatory proteins, such as, *SHINE1/Wax Inducer1* (*SHN1/WIN1*) in *Arabidopsis thaliana* (Aharoni et al., 2004; Broun et al., 2004), *WXP1/2* in *Medicago* (Zhang et al., 2005, 2007), *Outer Cell Layer 1* (*OCL1*) in *Zea mays* (Javelle et al., 2010), MYB96 in *Arabidopsis* and *Camelina sativa* (Seo et al., 2011; Lee et al., 2014), MYB94 in *Arabidopsis* (Lee and Suh, 2015b), MYB106 and MYB16 in *Arabidopsis* and *Torenia fournieri*, respectively (Oshima et al., 2013), have been shown to be associated with surface wax deposition. The *SHN1/WIN1* proteins belonging to the APETALA2/ETHYLENE RESPONSE FACTOR (AP2/ERF) family are well known for their diverse functions including regulating plant developmental processes and imparting stress tolerance. The transgenic *Arabidopsis* plants overexpressing *SHN1/WIN1* showed dark green, glossy leaves with approximately 4.5-fold increased accumulation of epicuticular waxes in stem and leaves (Broun et al., 2004; Aharoni et al., 2004). The *AtSHN1* overexpressers showed improved tolerance to abiotic stresses (Aharoni et al., 2004; Broun et al., 2004; Kannangara et al., 2007; Karaba et al., 2007a,b). In this study we generated transgenic mulberry plants constitutively expressing *AtSHN1* and examined its effect on epicuticular wax load, composition and its impact on cuticular water loss. *AtSHN1* overexpression increased leaf surface wax load and improved leaf MRC in mulberry.

## Materials and Methods

### Cloning of *SHN1* from *Arabidopsis thaliana*

Full length *SHN1* (AT1G15360)^1^ was isolated from the genomic DNA of *A. thaliana* using high-fidelity DNA polymerase (Finnzymes, Finland). Genomic DNA was isolated from tender leaves using the cetyltrimethyl ammonium bromide (CTAB) method (Muhammad et al., 1994). The polymerase chain reaction (PCR) was performed in a gradient PCR system (Mastercycler, Eppendorf, Germany) using *SHN1* specific forward and reverse primers (Supplementary Table 1). The amplified product was gel purified using GenElute^TM^ gel extraction kit (Sigma, USA) and cloned into T/A (pTZ57R/T) cloning vector (MBI Fermentas, Hanover, MD, USA) and sequence verified (ABI 3730; Applied BioSystems, Foster City, CA, USA).

### Construction of *AtSHN1* Overexpression Vector

The full-length *AtSHN1* was released from the pTZ57R/T:*AtSHN1* plasmid by *Sma1* and *Sac1* restriction enzymes and sub-cloned into binary vector pBI121. The recombinant overexpression construct designated as pBI121-*P_CaMV 35S_::AtSHN1:T_nos_* was mobilized into *Agrobacterium* strain EHA105 by electroporation (Sambrook et al., 1989) and used for transformation. *Agrobacterium* was cultured in AB minimal medium supplemented with kanamycin (50 mg L^-1^), rifampicin (10 mg L^-1^) and acetosyringone (200 μM) for 18–20 h at 28°C, at 230 rpm in dark. Bacterial culture in its early log phase (optical density at 600 nm of 0.6–0.8) was chosen for plant transformation.

### Mulberry Transformation and Regeneration

The seeds of mulberry, *Morus indica (*cv. M5) harvested from fresh fruits were surface sterilized with HgCl_2_ (0.1%, w/v) for 8 min and washed five to six times with sterile water. Seeds were imbibed overnight in sterile water were cultured on MS (Murashige and Skoog, 1962) medium in dark for 5–8 days before shifting to long-day culture conditions (16/8 h light/dark) at 26 ± 2°C. Hypocotyls and cotyledons excised from 15 days old seedlings were used as explants for plant transformation.

*Agrobacterium*-mediated transformation protocol established by Bhatnagar and Khurana (2003) was followed to generate transgenic mulberry plants with minor modifications. Hypocotyl and cotyledon explants were pre-incubated for 5 days on MS medium containing thidiazuron (TDZ) (0.1 mg L^-1^). After *Agrobacterium* infection, the explants were incubated on MS medium containing TDZ (1.1 mg L^-1^) and acetosyringone (250 mM) for 3 days in dark. Subsequently, the explants were cultured on MS medium containing TDZ (0.1 mg L^-1^), cefotaxime (200 mg L^-1^), and kanamycin (50 mg L^-1^). To select transformed tissue, selection pressure [kanamycin (50 mg L^-1^)] was applied for 45 days and healthy shoots were separated and transferred to rooting media containing indole butyric acid, (IBA, 0.5 and 1.0 mg L^-1^) in the presence (1.0%, w/v) or absence of activated charcoal. Well rooted plantlets were hardened on soilrite and healthy plantlets were transplanted into pots filled with potting mixture 2:1:1 (garden soil, sand, and farmyard manure), and allowed to grow in transgenic containment facility.

### Polymerase Chain Reaction (PCR) and RT-PCR Analysis

Genomic DNA was isolated from leaves of wild type and four transgenic lines (35S S–L1, L2, L3 and L4) using the CTAB method (Muhammad et al., 1994). To confirm the presence of genes, PCR was performed using *neomycin phosphotransferase II* (*NptII*) and *AtSHN1* gene-specific forward and reverse primers (Supplementary Table 1). Further, PCR was also performed using *AtSHN1* forward and Nos terminator reverse primers (Supplementary Table 1). The identity of the amplified product was confirmed by sequencing (ABI 3730; Applied BioSystems, Foster City, CA, USA).

For transgene expression analysis, total RNA was isolated from leaf tissues (100 mg) collected from wild type and four transgenic lines (35S S–L1, L2, L3, and L4) using the modified lithium chloride precipitation method by Sajeevan et al. (2014). All samples were treated with *DNase1* to remove genomic DNA contamination and about 4 μg of total RNA was used as the template to synthesize cDNA using the RevertAid First Strand cDNA Synthesis Kit (MBI Fermentas, Hanover, MD, USA). The first strand cDNA template was used to examine the expression of transgene using *AtSHN1* gene-specific primers (Supplementary Table 1). The house keeping gene *actin* (Supplementary Table 1) was used as an internal control for all the PCR reactions. The RT-PCR products were separated by agarose gel electrophoresis (Sambrook et al., 1989), documented using gel documentation system (Herolab, Germany) and product intensities were quantified using ImageJ 1.45s software^2^ and presented as relative expression.

### Southern Hybridization

Transgene integration was assessed by Southern blot hybridization. Genomic DNA (15 μg) was digested with *HindIII* restriction enzyme at 37°C overnight. The digested DNA was separated on 0.8% (w/v) agarose gel and transferred to positively charged Hybond-N+ nylon membrane (Amersham, UK). For Southern blot, *nptII* gene fragment (790 bp) was PCR amplified using binary vector pBI121 as template and labeled with Digoxigenin-11-dUTP using DIG-High Prime DNA labeling and detection kit (Roche Applied Science, catalog 11745832910) as per manufacturer’s instructions. Probe labeling strength was quantified as per kit instructions. Prehybridization of blot was carried out at 60°C for 4 h. Hybridization with denatured probe (∼1000 ng) was carried out overnight at 60°C. Post hybridization blot was washed twice for 15 min each at 60°C; once with 2X Saline-Sodium Citrate (SSC) and 1% (w/v) Sodium Dodecyl Sulphate (SDS) followed by another wash with 2X SSC and 0.5% (w/v) SDS. Probe hybridization was detected by anti-DIG antibody conjugated with alkaline phosphatase using substrates nitroblue tetrazolium chloride (NBT) and 5-bromo-4-chloro-3-indolyl-phosphate (BCIP) as per manufacturer’s instructions.

### Epicuticular Wax Quantification

Leaf surface waxes were extracted and quantified using a colorimetric method (Ebercon et al., 1977; Mamrutha et al., 2010). This method is based on the color change produced by the reaction of wax with acidic potassium dichromate (K_2_Cr_2_O_7_). Carnauba wax (Sigma, USA) was used as a standard for leaf surface wax quantification (Samdur et al., 2003). Total leaf surface wax amount was expressed as μg dm^-2^.

### Scanning Electron Microscopy (SEM)

Scanning electron microscopy was used to study surface wax morphology on the adaxial and abaxial surfaces of mature leaves collected from two selected mulberry transgenic lines (35S S-L2 and L4) and wild type plant. Leaf tissue was fixed in 5% (v/v) glutaraldehyde and mounted on stubs. Samples were coated with gold particles for 10 min. Coated samples were transferred to an ESEM, Quanta 200 (FEI, USA) scanning electron microscope for examination (Chuong et al., 2006).

### Wax Extraction and Analysis of Wax Composition

Leaf surface wax was extracted from mature leaves of two selected mulberry transgenic lines (35S S-L2 and L4) and wild type plant using chloroform for 15 s (Mamrutha et al., 2010). The leaf surface extracts contained waxes from both adaxial and abaxial leaf surfaces. Chloroform was evaporated and the dried wax was dissolved in hexane and injected to gas chromatograph–mass spectrometry (GC-MS) for analysis of the profile (Gutierrez et al., 1998). The analysis was performed on a Varian-3800 gas chromatograph coupled with Varian 4000 GC-MS/MS (Varian, USA) ion-trap mass selective detector. Wax compounds were separated on DB-5MS (Varian, USA) column (30 m × 0.25 mm i.d. with 0.25 μm film thickness) using the temperature program with injector port temperature at 300°C, column temperature program of 100°C for 2 min; increasing at 6°C/min to 244°C, 2 min at 244°C; increasing at 8°C/min to 300°C, and 30 min at 300°C. Wax composition was determined by comparing peak retention times with those of reference standards (Pentadecane), and by a GC-MS analysis of representative samples. The mass spectrometer was operated in the external electron ionization mode with the carrier gas helium 1 ml/min.; injector temperature, 300°C; trap temperature 200°C, ion source-heating at 200°C and transfer line temperature 300°C, EI-mode was 70 eV, with the full scan-range 50–450 amu.

### Leaf Surface Hydrophobicity

Contact angle, a measure of surface hydrophobicity, was measured by the contact angle goniometer method (Yuan and Lee, 2013). The measurements were made using demineralized deionized water droplets. Leaf disks were collected and placed on the measuring platform with double sided tape as adhesive. A known volume (15 μl) of droplet was pointed vertically down onto the sample surface and the contact angle was captured with a high resolution camera (Olympus – B061, Japan) having a protractor mounted in the eye-piece. Diameter of the drop formed on the leaf is measured using a vertical stereoscope microscope, employing the stage and ocular micrometer. The diameter of water drop on the leaf is measured in two ways viz., north–south and east–west using the ocular micrometer and then expressed in millimeter (mm). All measurements were made under laboratory conditions at temperature 25 ± 1°C and relative humidity (RH) of 50 ± 5%.

### Chlorophyll Leaching Assay

For chlorophyll leaching assay, mature leaves were collected and rinsed with tap water, weighed, and put in tubes containing 20 mL of ethanol (80%, v/v) at room temperature (gently agitating in the dark). The amount of chlorophyll extracted into the solution was estimated every 30 min upto 5 h after recording the absorbance at wavelengths 663 and 645 nm using a spectrophotometer (SPECTRA max PLUS 384, Molecular devices; Hiscox and Israelstam, 1979).

### Moisture Retention Capacity (MRC)

Leaves were harvested early in the morning and fresh weight recorded immediately. Leaf weight was recorded using an electronic balance with precision of 0.1 mg (Sartorius, Gottingen, Germany) at hourly intervals up to 5 h (Mamrutha et al., 2010). The experiments were conducted at constant temperature (30 ± 0.5°C) and RH (55–60%) under a light intensity of 500–550 mmol m^-2^ s^-1^. At end of the experiment, leaves were dried to a constant weight in a hot air oven at 80°C for 24 h. The MRC was estimated using the formula:

$$\begin{array}{c} \mathrm{MRC}(\%)\;=\;\{({\mathrm{FW}}_{1}\;-\;\mathrm{DW})/({\mathrm{FW}}_{0}\;-\;\mathrm{DW})\}\;\times \;100 \end{array}$$

where, FW_o_ is the fresh weight (g) immediately after harvest, FW_1_ is the weight (g) at a particular hour after harvest and DW is the oven dry weight (g).

### Soluble Protein Content

The content of soluble protein was estimated from the leaf samples at different time points post-harvest following the method of Bradford (1976) and expressed as mg g^-1^ fresh weight. The leaf sample of 0.1 g was macerated in 10 mL of phosphate buffer (0.1 M, pH 7.0) using a pestle and mortar. The color intensity in the protein extract after the reaction with reagent was recorded at wavelength 595 nm using a spectrophotometer (SPECTRA max PLUS 384, Molecular devices). Bovine serum albumin (BSA) was used as standard.

### Silkworm Bioassay

Hybrid (PM × CSR2) bivoltine 5th larval instar worms of *B. mori* L. were procured from the College of Sericulture, University of Agricultural Sciences, Chintamani, Karnataka. Silkworms were reared on the fresh tender leaves of the *AtSHN1* transgenic mulberry lines and wild type leaves at 24–28°C under a 16/8-h (light/dark) photoperiod under controlled environment conditions according to the protocol of Kawakami and Yanagawa (2003). Seventy larvae’s were used for each treatment, fed thrice a day (at 8.00 AM, 2.00 and 8.00 PM). The molting larvae were mounted on bamboo mountage at the rate of 50 worms per square feet per treatment and cocoons were harvested after 5 days. The spacing of larvae and other rearing requirements were practiced as recommended by Sekharappa et al. (1991). Increase in weight of 5th instar larvae was recorded from day 1 of rearing (gm). After complete mounting cocoon weight, shell weight and pupae weight were recorded (gm) and the effective rate of rearing (ERR) was calculated in percentage (%) using the formula:

$$\begin{array}{c} \mathrm{ERR}(\%)\;=\;(Na/Nb)\;\times \;100 \end{array}$$

where, Na is number of cocoons obtained and Nb is number of worms brushed.

### Statistical Analysis

All the experiments were conducted in three biological replicates unless otherwise mentioned and SE was computed in each case. For the estimation of statistical significance, Student’s *t-*test was performed. The data points representing statistically significant differences between wild type and transgenic lines have been indicated.

## Results

### *Agrobacterium* Mediated Transformation and Generation of Transgenic Plants

**FIGURE 1:**
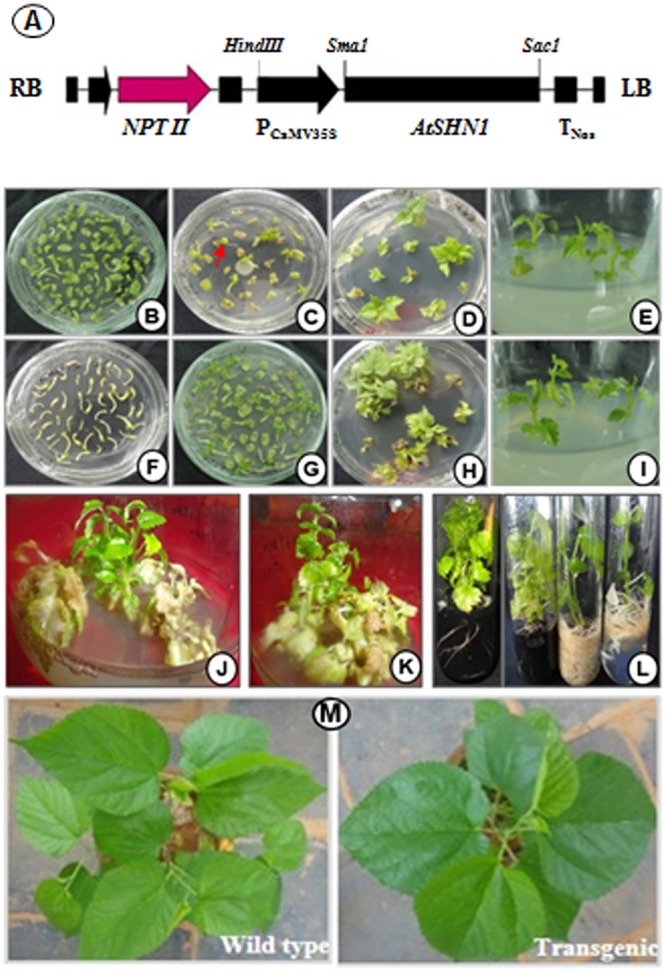
**Genetic transformation and development of mulberry transgenic plants to express *AtSHN1.* (A)** schematic representation of T-DNA region of pBI121 carrying *AtSHN1*gene; **(B)** cotyledons separated from seedlings in pre-incubation medium; **(C)** emergence of shoots after the infection with *Agrobacterium* in selection medium with kanamycin 50 mg L^-1^; **(D,E)** regeneration of individual events in growth medium containing kanamycin 50 mg L^-1^; **(F)** hypocotyls separated from seedlings in pre-incubation medium; **(G)** emergence of shoots after the infection with *Agrobacterium* in selection medium with kanamycin 50 mg L^-1^; **(H,I)** growth of select transgenic lines in selection medium; **(J,K)** stringent screeing of transgenic lines in selection medium (kanamycin 50 mg L^-1^); **(L)** well rooted putative mulberry transgenics with and without activated charcoal; **(M)** 2 month old mulberry transgenic plant showing deep green, shiny appearance compared to wild type.

Full length of *AtSHN1* (1135 bp) amplified from *A. thaliana* genome confirmed by sequencing (data not shown) was used for the construction of overexpression vector (**Figure 1A**). To generate transgenic mulberry through *in vitro* transformation approach, explants were pre-cultured on MS medium containing TDZ (0.1 mg L^-1^) for 5 days (**Figures 1B,F**) and infected with *Agrobacterium* carrying the recombinant plasmid. Since bacterial cell density, infection time and co-cultivation duration are the important factors for transformation experiments, experimental conditions were standardized initially. Three days of co-cultivation in dark was found to be essential for infection without any negative effects on explants. Culturing the explants in a selection medium containing TDZ (0.1 mg L^-1^), cefotaxime (200 mg L^-1^), and kanamycin (50 mg L^-1^) for 45 days with a subculture at every 15 days interval yielded satisfactory results. Initially, 30 days post inoculation, nodules-like structures were noticed in the midrib region and basal cut ends. These structures later turned into shoot buds and subsequently regenerated into shoots (**Figures 1C–E,G–I**). Healthy shoots of 5–7 cm length (4–5 leaf stage) were separated (**Figures 1J,K**) and transferred to rooting media containing full or half strength MS and IBA (0.5 and 1.0 mg L^-1^) with or without activated charcoal (1.0%, w/v) (**Figure 1L**). Well rooted plantlets showed 80–90% survival. The putative transgenic plants showed deep green and shiny phenotype compared to wild type plants under normal growth conditions (**Figure 1M**).

### Molecular Characterization of Transgenic Plants

**FIGURE 2:**
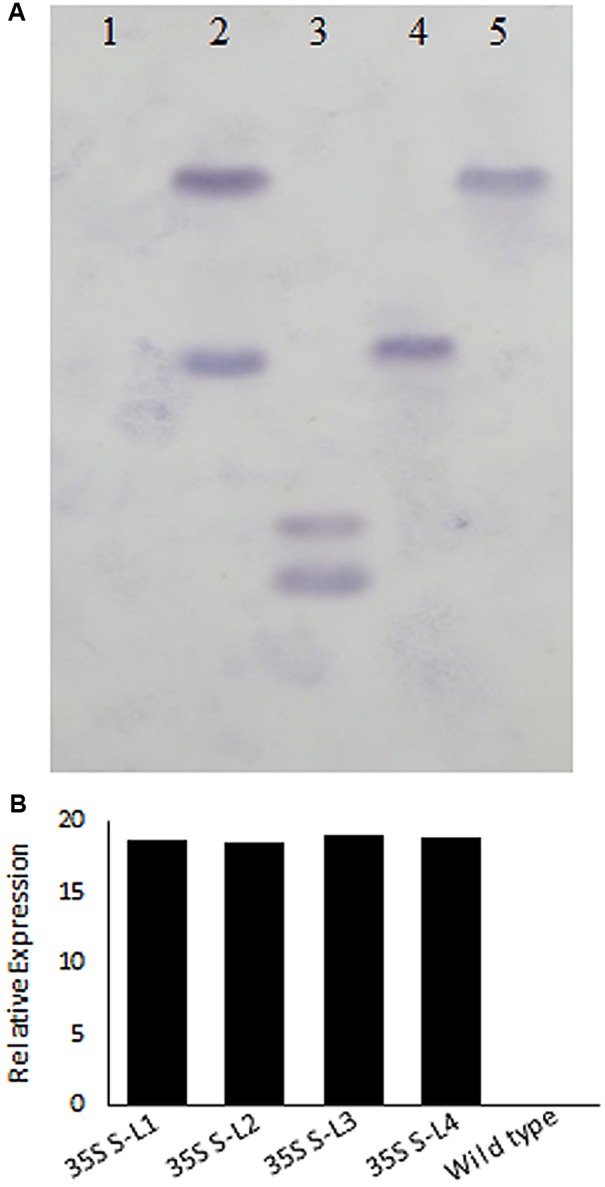
**Molecular characterization of transgenic and wild type mulberry plants. (A)** Southern blot analysis using genomic DNA of transgenic and wild type plants. 15 μg genomic DNA each from putative transgenic and wild type (untransformed) plants was digested with *HindIII* and probed with labeled *NptII* PCR product. Lane 1 is wild type (untransformed plant), Lanes 2–5 are different mulberry transgenic lines. **(B)** Relative expression of *AtSHN1* gene in transgenic and wild type mulberry plants. 35S S-L1, L2, L3, and L4 are different transgenic lines.

Polymerase chain reaction analysis carried out using *nptII* and *AtSHN1* specific primers confirmed the integration of T-DNA into the mulberry genome (Supplementary Figures 1A,B). PCR with *AtSHN1* specific forward primer and Nos Terminator reverse primer yielded 1460 bp product which was eluted and sequenced (Supplementary Figures 1C,D). Southern hybridization carried out using probe specific to *nptII* (**Figure 2A**) showed the integration of T-DNA in the genome of transgenic lines (**Figure 2A**). Expression of *AtSHN1* in transgenic lines assayed by RT-PCR indicated expression of transgene (**Figure 2B**).

### Epicuticular Wax Load and Scanning Electron Microscopy (SEM)

**FIGURE 3:**
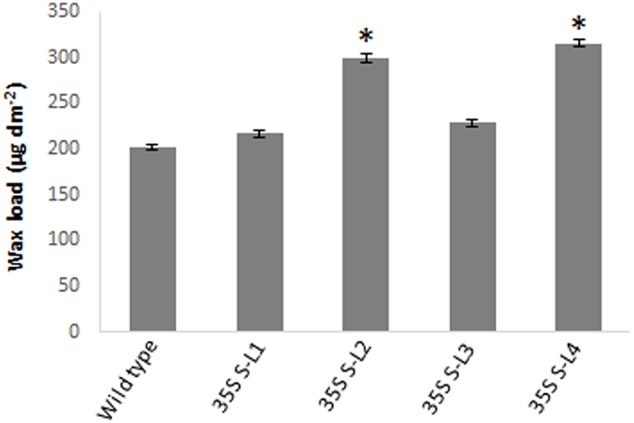
**Estimation of total surface wax load from leaves of 4 months old transgenic and wild type mulberry plants grown under normal conditions.** Error bars indicate ±SE and statistical significance were shown with asterisk (*P* < 0.05). 35S S-L1, L2, L3, and L4 are different transgenic lines.

**FIGURE 4:**
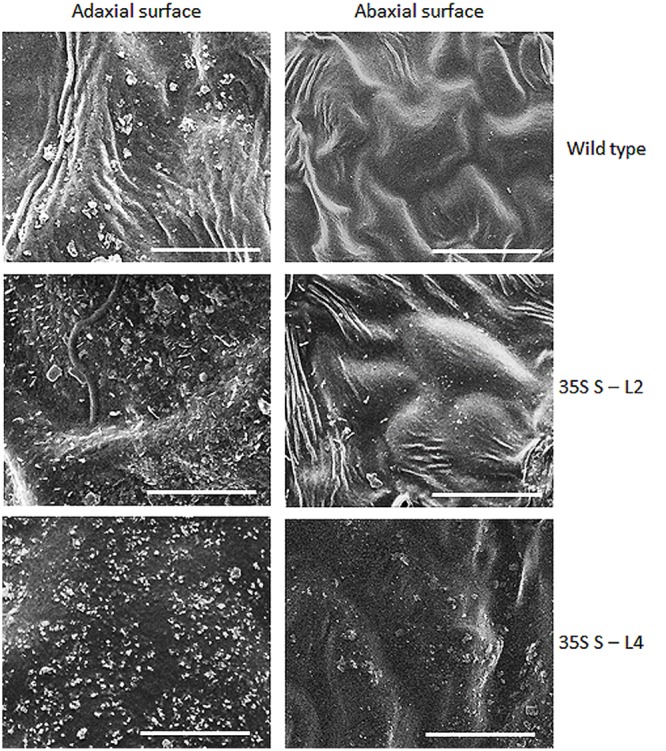
**Analyses of wax crystals morphology in adaxial and abaxial sides of mulberry transgenics and wild type plants using scanning electron microscopy (SEM).** The scale is 10 μm with images taken at 5,000× magnification.

Significant difference (*P <* 0.05) was observed in total wax load between transgenic and wild type plants. Total wax load was 0.75 to 1.2-fold higher in *AtSHN1* overexpression lines compared to wild type plants (**Figure 3**). SEM analysis showed difference in epicuticular wax crystal morphology between transgenic and wild type mulberry plants. Expression of *AtSHN1* changed the pattern of epicuticular wax crystals on the adaxial and abaxial leaf surfaces. Compared to adaxial leaf surface, there were fewer wax crystals observed in abaxial surface of mulberry transgenic plants (**Figure 4**).

### Analysis of Epicuticular Wax Components

**FIGURE 5:**
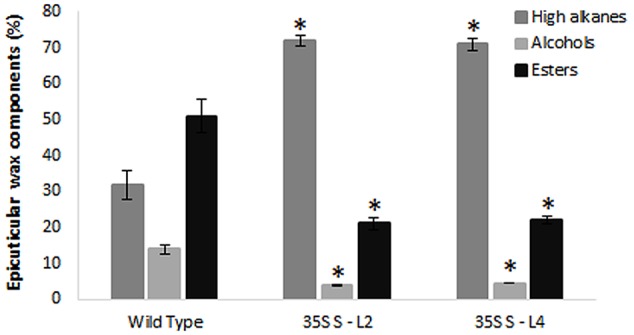
**Analyses of leaf wax components in transgenics and wild type mulberry plants.** Wax constituents of higher alkanes, alcohols, and esters in leaves are expressed as percent of total wax. The results show averages of three replicates and statistical significance were shown with asterisk (*P* < 0.05) and error bars indicate mean ± SE.

Wax components in leaf samples were analyzed by GC-MS in selected transgenic mulberry lines (S-L2 and S-L4). There were significant differences (*P <* 0.05) in wax components between the wild type and transgenic mulberry plants. Higher alkanes were 2.2-fold more (72 and 71%) in mulberry *AtSHN1* overexpressors compared to wild type plants (32%) whereas alcohols and esters were significantly reduced with 3.2 and 2.5-fold reduction in alcohol (3.9 and 4.4%) and ester levels (21 and 21.9%) compared to wild type plants (14 and 51%) respectively (**Figure 5**).

### Leaf Surface Properties and Cuticular Permeability

**FIGURE 6:**
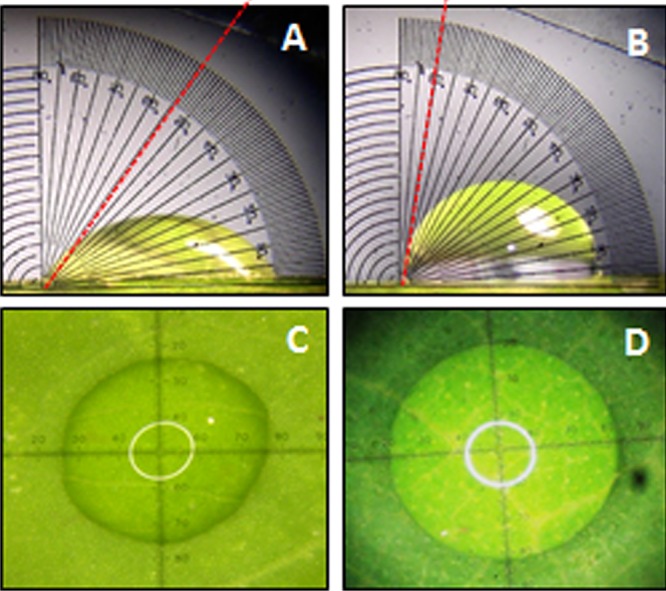
**Measurement of water drop characteristics on leaves of mulberry transgenics and wild type plants. (A,B)** Contact angle of wild type and *AtSHN1* overexpressors, red doted line shows the contact angle of droplets; **(C,D)** droplet diameter of wild type and *AtSHN1* overexpressors.

**FIGURE 7:**
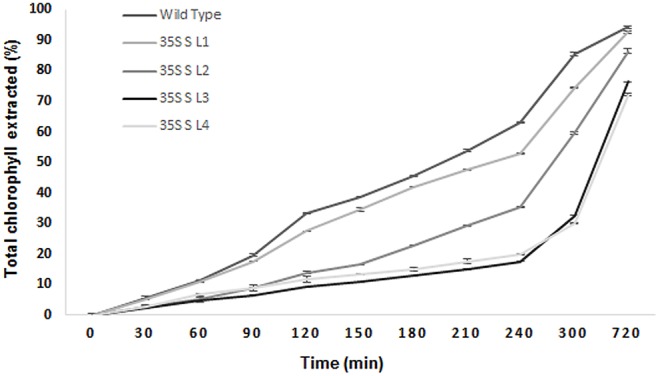
**Chlorophyll leaching assay using leaves collected from wild type and transgenic mulberry plants at different time points.** The results show averages of three replicates, 35S S-L1, L2, L3, and L4 are different transgenic lines.

A significant difference (*P <* 0.05) in extent of hydrophobicity was observed between transgenic and wild type mulberry leaf surfaces (**Table 1**). Lower contact angle of water droplets in wild type (55°), than transgenic lines (72–81°) indicated changes in leaf surface properties (**Table 1** and **Figures 6A,B**). Similarly, we noticed differences in droplet diameter between wild type and transgenic lines (**Table 1** and **Figures 6C,D**). Chlorophyll leaching assay was carried out to test the cuticular membrane permeability in fully mature leaves. Significantly higher (*P <* 0.05) quantities of chlorophyll could be extracted from wild type compared to transgenic lines suggesting higher cuticular resistance for chlorophyll leaching in transgenic lines (**Figure 7**).

**Table 1:** Contact angle and droplet diameter of mulberry transgenics and wild type plants.

Sl. No.	Name	Contact angle (°)	Droplet diameter (mm)
(1)	Wild type	56 ± 1.20	4.61 ± 0.46
(2)	35S S-L1	79 ± 1.60^∗^	3.70 ± 0.29
(3)	35S S-L2	72 ± 1.13^∗^	3.84 ± 2.19
(4)	35S S-L3	74 ± 0.91^∗^	3.72 ± 0.92
(5)	35S S-L4	81 ± 0.75^∗^	3.17 ± 0.98^∗^

*The values show average of five replications and mean ± SE. Asterisk indicates statistical significance (*P* < 0.05).*

### Moisture Retention Capacity and Soluble Protein Content

**FIGURE 8:**
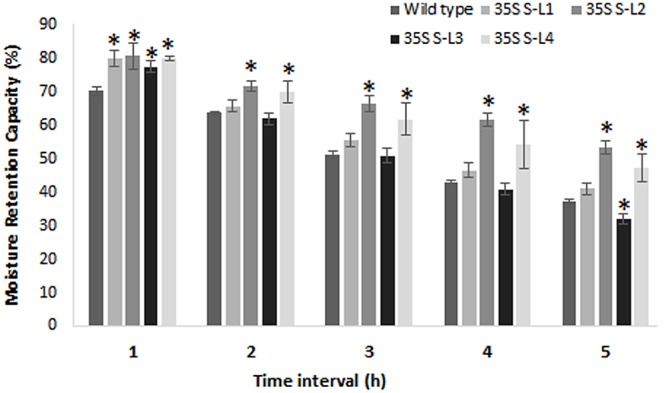
**Estimation of moisture retention capacity of leaves of 4 months old transgenic and wild type mulberry plants.** Error bars indicate mean ± SE and statistical significance of differences between wild type and transgenic plants are indicated with asterisk (*P* < 0.05). 35S S-L1, L2, L3, and L4 are different transgenic lines.

**FIGURE 9:**
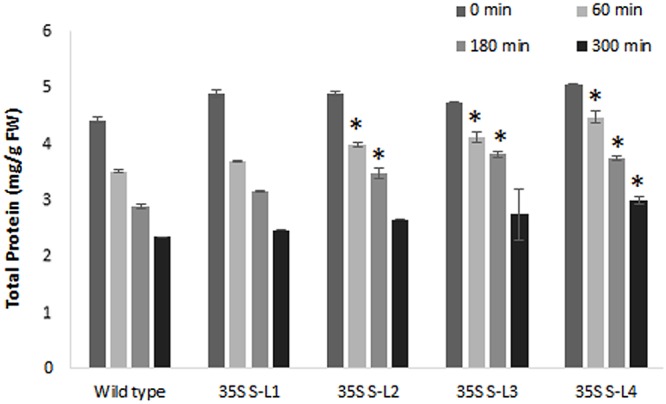
**Estimation of total soluble protein content from detached leaves of 4 months old transgenic and wild type mulberry plants at different time intervals.** Error bars indicate mean ± SE and statistical significance was shown with asterisk (*P* < 0.05). 35S S-L1, L2, L3, and L4 are different transgenic lines.

Transgenic mulberry plants expressing *AtSHN1* showed significant reduction in post-harvest water loss through cuticle compared to wild type plants. Transgenic lines maintained higher leaf moisture content (48–54%) compared to wild type (37%), even after 5 h of harvest (**Figure 8**). To compare the beneficial effect of the higher MRC in transgenic plants, we quantified the total buffer soluble protein in the harvested leaves. Three *AtSHN1* overexpression lines (35S S–L2, L3, and L4) showed delay in protein degradation as indicated by higher protein content at 1, 3, and 5 h post-harvest compared to wild type plants (**Figure 9**), which might be due to slow protein degradation post-harvest.

### Silkworm Bioassay

**FIGURE 10:**
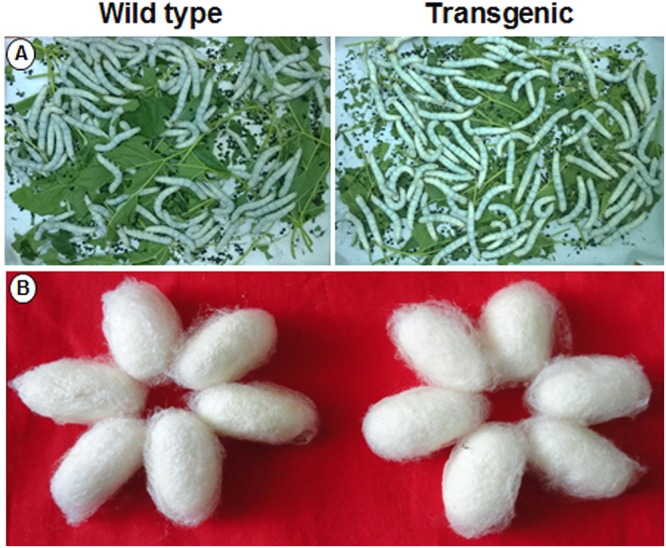
**Silkworm bioassay.** Representative pictures showing **(A)** hybrid bivoltine 5th instar larvae feeding on transgenic and wild type mulberry leaves; **(B)** morphology of the cocoons harvested.

A silkworm bioassay was conducted to study the effect of the transgene protein on *B. mori* larvae, feeding and rearing. Young tender leaves from wild type and transgenic plants were fed to the 5th instar larvae thrice a day (**Figure 10A**). An increase in larvae’s weight was observed daily, but there was no significant difference noticed between the larvae fed with wild type and transgenic plant leaves (Supplementary Table 2A). There was no significant difference in cocoon weight between wild type and transgenic treatments (**Figure 10B** and Supplementary Table 2B). There was an increase in shell, pupal weight and ERR of silkworms fed with transgenic lines compared to wild type (Supplementary Table 2B).

## Discussion

Leaf surface properties, wax content and wax crystal morphology influence post-harvest water loss in mulberry (Mamrutha et al., 2010). Plant cuticle plays an important role in limiting water loss as demonstrated in many different studies (Schreiber and Riederer, 1996; Jenks and Ashworth, 1999; Riederer and Schreiber, 2001; Jenks et al., 2002). Targeted manipulation of cuticular wax biosynthetic pathway can be a viable option to increase surface wax in plants. Many TFs associated with wax production have been identified and their relevance demonstrated in plants (Broun et al., 2004; Karaba et al., 2007a,b; Seo et al., 2011; Lam et al., 2012; Yeats and Rose, 2013). Many earlier studies have shown that *AtSHN1* overexpressing plants exhibit altered epidermal properties resulting in tolerance to dehydration stress (Aharoni et al., 2004; Kannangara et al., 2007). In an attempt to reduce the post-harvest water loss by altering the leaf surface wax load in mulberry, we expressed *AtSHN1* gene cloned from *Arabidopsis* in mulberry under a constitutive CaMV35S promoter.

Over 1.2-fold increase in total wax content in transgenic lines suggests that *AtSHN1* expression activated wax biosynthesis and secretion pathways in mulberry. A strong positive relationship between surface wax load and MRC of harvested leaves of mulberry as reported by Mamrutha et al. (2010) suggest that water loss can be minimized by altering surface waxes. The permeability barrier properties of cuticle are mainly determined by the aliphatic constituents of the cuticular waxes (Vogg et al., 2004). The increase in amount of higher alkanes and decline in alcohol and ester levels in *AtSHN1* overexpressors, accompanied by an increase in cuticular resistance seen in the mulberry transgenic plants signifies the importance of alkanes in determining MRC. A disproportionate build-up of higher alkanes, in contrast with the reduction in alcohol and ester levels in mulberry *SHN1* overexpressors, suggests a preferential increase in flux through the decarbonylation pathway, than the acyl reduction pathway. By contrast to our study, the *shn* mutants in *Arabidopsis* showed a sharp increase in alkanes, primary and secondary alcohols, alkyl esters, ketones, and aldehydes resulting from both the decarbonylation and acyl reduction pathways (Aharoni et al., 2004). There are evidences to indicate that different wax biosynthesis genes or their homologs from other distant plant species contribute for the variations in wax compositions (Chen et al., 2003; Sturaro et al., 2005; Zhang et al., 2005; Wang et al., 2012). For example, *SHN1/WIN1* in *Arabidopsis* increases cuticular wax load by mainly altering alkane content (Aharoni et al., 2004; Broun et al., 2004), where as in *Medicago* primary alcohol, a predominant wax component, is probably regulated by *WXP1* (Zhang et al., 2005). It has also been reported that alkanes are predominantly present in the mature leaves of *Arabidopsis* (Suh et al., 2005; Seo et al., 2011), maize (Blaker and Greyson, 1988), tree tobacco (Cameron et al., 2006), tomato (Leide et al., 2007). In the present study, an increase in alkane concentration was observed in *AtSHN1* transgenic plants and the shift in wax composition may lead to changes in overall surface wax crystallinity in mulberry leaves.

Since droplet contact angle and droplet diameter studies help in understanding the properties of leaf surface, we measured this parameter. Leaf is considered hydrophilic when it forms a contact angle less than 90° and nearer or greater than 90° considered as hydrophobic (Bhushan, 2003; Bhushan et al., 2004). Transgenic lines showed more contact angle and less droplet diameter (spreading) compared to wild type plants. Rice mutants for *Wax crystal-sparse leaf2* (*wsl2*), displayed more droplet spreading, unlike water droplets forming beads in wild type (Mao et al., 2012). Cuticle permeability is strongly influenced by the quantity and composition of cuticular wax present. Chlorophyll efflux/leaching assay showed significant reduction in chlorophyll extracted from mulberry *AtSHN1* overexpressors compared to wild type plants, consistent with observations of reduced post-harvest water loss. Our results were contradictory with the results of Aharoni et al. (2004) in *Arabidopsis*, and is in agreement with the findings of Wang et al. (2012), who demonstrated that the expression of *OsWR1*, a homolog of *AtWIN1/SHN1*, reduces chlorophyll leaching and water loss from dissected leaves of rice while RNA interference (RNAi) of *OsWR1* increases chlorophyll leaching and water loss. In our study, *AtSHN1* expression resulted in significant improvement in moisture retention ability in comparison to wild type plants even after 5 h post-harvest. Higher MRC of harvested leaves may contribute for the maintenance of better leaf quality for a longer period, which was evident in our study as there was higher buffer soluble protein content in leaves at different time points post-harvest in transgenic lines when compared to wild type plants.

To examine the effect of *AtSHN1* overexpression on silkworm growth and cocoon parameters, we carried out silkworm bioassay. Our silkworm bioassay led to the conclusion that over production of *SHN1* protein and associated phenotypic changes, (increase in epicuticular wax content) has no deleterious effect on the growth and feeding behavior of silkworm larvae. The cocoon produced from the silkworms fed with transgenic mulberry leaves did not show any difference in color or texture (**Figure 10B**). Similar results were also reported by Lal et al. (2008) and Das et al. (2011) in silkworm rearing studies using the transgenic mulberry leaves expressing tobacco *osmotin* and barley *HVA1* genes. Since commercial sericulture involves indoor rearing of silkworms using harvested mulberry leaves, any improvement in MRC, as demonstrated in this study can contribute for leaf quality and hence cocoon yield. In summary, the study demonstrated that overexpression of *AtSHN1* gene in mulberry can enhance the total wax load, alter leaf surface properties and help in delaying post-harvest water loss. The study also demonstrated that overexpression of upstream regulatory gene/s associated with specific trait/s can be viable approach for targeted crop improvement in perennial tree crops such as mulberry.

## Data Archiving Statement

Gene sequence used in this study was reported earlier in “The Arabidopsis Information Resource (TAIR) database” and the accession number of the gene is given in the section “Materials and Methods.”

## Author Contributions

Conceived and designed the experiments: KN and RS. Performed the experiments: RS, KS, NP, and DG. Analyzed the data: RS, KS, and KN. Contributed reagents/materials/analysis tools: KN. Wrote the paper: RS, KN, DG, and MS.

## Conflict of Interest Statement

The authors declare that the research was conducted in the absence of any commercial or financial relationships that could be construed as a potential conflict of interest.

## Footnotes

1 http://www.arabidopsis.org

2 http://imagej.nih.gov/ij
