# Supplementary material for: Expression of Arabidopsis SHN1 in Indian Mulberry (Morus indica L.) Increases Leaf Surface Wax Content and Reduces Post-harvest Water Loss
Source: Front Plant Sci. 2017 Apr 4;8:418. doi: 10.3389/fpls.2017.00418 (PMC5378817; doi:10.3389/fpls.2017.00418)
Supplement: Supplementary file 1 [file Table_1.PDF]

Supplementary Table I. List of primers used in the present study.

Sl. No.	Primer Name	Sequence 5´-3´	Purpose
1a.	*AtSHN1* F	CCATCTTACATATATTACTCATCATC	Amplification of *SHINE1* full-length gene and confirmation of transgenic plants
1b.	*AtSHN1* R	GTACAAACACCAATACTTTATTAGATAC	
2a.	*NptII* F	GAGGCTATTCGGCTATGACTG	Confirmation of transgenic plants
2b.	*NptII* R	GATACCGTAAAGCACGAGG	
3a.	Nos Terminator R	GATCTAGTAACATAGATGAC	Confirmation of transgenic plants
3b.	*AtSHN1* F	CCATCTTACATATATTACTCATCATC	
4a.	RT *AtSHN1* F	GACCGCAGAGGAGGCAGC	Expression confirmation of *SHINE1* gene
4b.	RT *AtSHN1* R	GCCGATATGGGAGCTGGCTG	
5a.	RT *Actin* F	TCCATAATGAAGTGTGATGT	Internal control for expression analysis
5b.	RT *Actin* R	GGACCTGACTCGTCATACTC	
