# Supplementary material for: Expression of Arabidopsis SHN1 in Indian Mulberry (Morus indica L.) Increases Leaf Surface Wax Content and Reduces Post-harvest Water Loss
Source: Front Plant Sci. 2017 Apr 4;8:418. doi: 10.3389/fpls.2017.00418 (PMC5378817; doi:10.3389/fpls.2017.00418)
Supplement: Supplementary file 2 [file Table_2.pdf]

Supplementary Table 2. Silkworm bioassay with *AtSHN1* transgenic as well as wild type plant leaves.

A. Weight of 5th instar larvae observed during rearing in days.

	**Weight of 5th instar larvae from day one during rearing (g)**						
	**1**	**2**	**3**	**4**	**5**	**6**	**7**
**Wild type**	30.1	35.6	42.4	48.6	54.1	61.1	67.5
***AtSHN1* transgenic**	30.8	36.2	40.8	48.2	53.6	60.4	67.6

B. Different parameters such as weight of cocoon, weight of shell, weight of pupa and effective rate of rearing (ERR) was recorded.

	**Weight of cocoon (g)**	**Weight of shell (g)**	**Weight of pupa (g)**	**ERR (%)**
**Wild type**	1.61	0.505	1.28	91.43
***AtSHN1* transgenic**	1.58	0.515	1.32	92.86

The silkworm bioassay experiment was carried out only once.
