# Supplementary material for: Expression of Arabidopsis SHN1 in Indian Mulberry (Morus indica L.) Increases Leaf Surface Wax Content and Reduces Post-harvest Water Loss
Source: Front Plant Sci. 2017 Apr 4;8:418. doi: 10.3389/fpls.2017.00418 (PMC5378817; doi:10.3389/fpls.2017.00418)
Supplement: Supplementary file 3 [file Presentation_1.PDF]

Supplementary Figure 1 - Molecular characterization of mulberry transgenic lines by PCR

A. *Neomycin phosphotransferase II* (*NptII*) specific forward and reverse primers

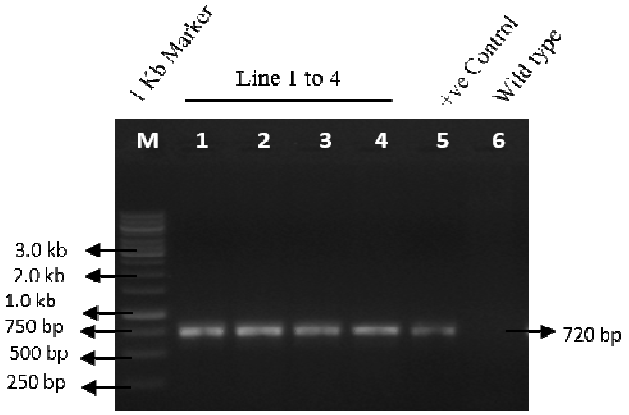

B. *AtSHN1*specific forward and reverse primers

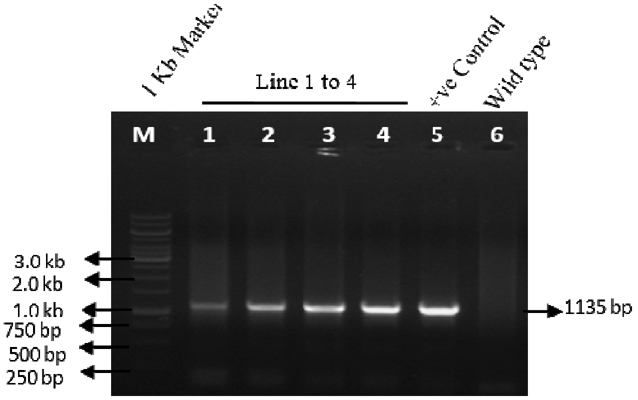

C. *AtSHN1*specific forward and Nos Terminator reverse primers

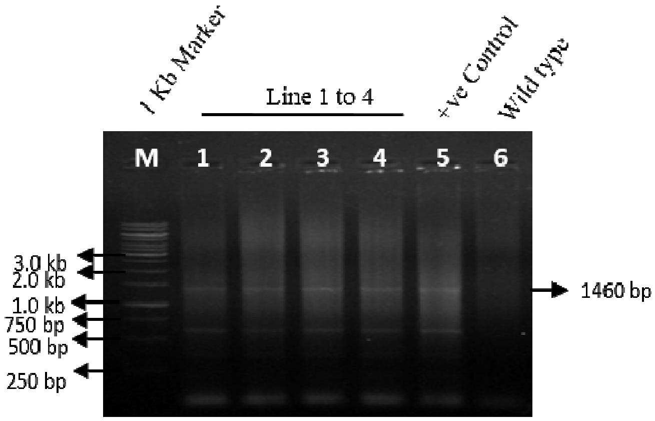

D. Multiple sequence alignment of sequences cloned from transgenic plants and plasmid (positive control) using San Diego Supercomputer Center (SDSC), Biology Workbench (http://workbench.sdsc.edu/).

```
CLUSTAL W (1.81) multiple sequence alignment

AtSHN1_NosTer_Cloned_1              TTTATCCTAGTTTGCGCGCTATATTTTGTTTTCTATCGCGTATTAAATGT
AtSHN1_NosTer_Cloned_2_             --TATCCTAGTTTGCGCGCTATATTTTGTTTTCTATCGCGTATTAAATGT
AtSHN1_NosTer_Vector_Control        -------TAGTTTGCGCGCTATATTTTGTTTTCTATCGCGTATTAAATGT
                                           *******************************************

AtSHN1_NosTer_Cloned_1              ATAATTGCGGGACTCTAATCATAAAAACCCATCTCATAAATAACGTCATG
AtSHN1_NosTer_Cloned_2_             ATAATTGCGGGACTCTAATCATAAAAACCCATCTCATAAATAACGTCATG
AtSHN1_NosTer_Vector_Control        ATAATTGCGGGACTCTAATCATAAAAACCCATCTCATAAATAACGTCATG
                                    **************************************************

AtSHN1_NosTer_Cloned_1              CATTACATGTTAATTATTACATGCTTAACGTAATTCAACAGAAATTATAT
AtSHN1_NosTer_Cloned_2_             CATTACATGTTAATTATTACATGCTTAACGTAATTCAACAGAAATTATAT
AtSHN1_NosTer_Vector_Control        CATTACATGTTAATTATTACATGCTTAACGTAATTCAACAGAAATTATAT
                                    **************************************************

AtSHN1_NosTer_Cloned_1              GATAATCATCGCAAGACCGGCAACAGGATTCAATCTTAAGAAACTTTATT
AtSHN1_NosTer_Cloned_2_             GATAATCATCGCAAGACCGGCAACAGGATTCAATCTTAAGAAACTTTATT
AtSHN1_NosTer_Vector_Control        GATAATCATCGCAAGACCGGCAACAGGATTCAATCTTAAGAAACTTTATT
                                    **************************************************

AtSHN1_NosTer_Cloned_1              GCCAAATGTTTGAACGATCGGGGAAATTCGAGCTCGGTACCTCGCGAATG
AtSHN1_NosTer_Cloned_2_             GCCAAATGTTTGAACGATCGGGGAAATTCGAGCTCGGTACCTCGCGAATG
AtSHN1_NosTer_Vector_Control        GCCAAATGTTTGAACGATCGGGGAAATTCGAGCTCGGTACCTCGCGAATG
                                    **************************************************

AtSHN1_NosTer_Cloned_1              CATCTAGATTTAGTTACAAACACCAATACTTTATTAGATACAAATGTGGA
AtSHN1_NosTer_Cloned_2_             CATCTAGATTTAGTTACAAACACCAATACTTTATTAGATACAAATGTGGA
AtSHN1_NosTer_Vector_Control        CATCTAGATTTAGTTACAAACACCAATACTTTATTAGATACAAATGTGGA
                                    **************************************************

AtSHN1_NosTer_Cloned_1              AAAAGAGAAACAATATATAGCTAACAACCTTTTAATATAAAACATCTATA
AtSHN1_NosTer_Cloned_2_             AAAAGAGAAACAATATATAGCTAACAACCTTTTAATATAAAACATCTATA
AtSHN1_NosTer_Vector_Control        AAAAGAGAAACAATATATAGCTAACAACCTTTTAATATAAAACATCTATA
                                    **************************************************

AtSHN1_NosTer_Cloned_1              AAATACACGGTATAATTGATTATTTTGGCTGTAAATCAGCAATGAAAATA
AtSHN1_NosTer_Cloned_2_             AAATACACGGTATAATTGATTATTTTGGCTGTAAATCAGCAATGAAAATA
AtSHN1_NosTer_Vector_Control        AAATACACGGTATAATTGATTATTTTGGCTGTAAATCAGCAATGAAAATA
                                    **************************************************

AtSHN1_NosTer_Cloned_1              GGTACATATATATAAGCAAATAAGATT-AGTTTGTATTGAGAAGCTCTTC
AtSHN1_NosTer_Cloned_2_             GGTACATATATATAAGCAAATAAGATTTAGTTTGTATTGAGAAGCTCCTC
AtSHN1_NosTer_Vector_Control        GGTACATATATATAAGCAAATAAGATTTAGTTTGTATTGAGAAGCTCCTC
                                    *************************** ******************* **

AtSHN1_NosTer_Cloned_1              TATCATTTGCAAAGCAACCTTTTCTTCCTCATCCAATACTTCTTCTCTGC
AtSHN1_NosTer_Cloned_2_             TATCATTTGCAAAGCAACCTTTTCTTCCTCATCCAATACTTCTTCTCTGC
AtSHN1_NosTer_Vector_Control        TATCATTTGCAAAGCAACCTTTTCTTCCTCATCCAATACTTCTTCTCTGC
                                    **************************************************

AtSHN1_NosTer_Cloned_1              TGCCACCAATTTCAACTTCAGTGGTCGGAGCAAGAATAGCGTCTTGTGAA
AtSHN1_NosTer_Cloned_2_             TGCCACCAATTTCAACTTCAGTGGTCGGAGCAAGAATAGCGTCTTGTGAA
AtSHN1_NosTer_Vector_Control        TGCCACCAATTTCAACTTCAGTGGTCGGAGCAAGAATAGCGTCTTGTGAA
                                    **************************************************

AtSHN1_NosTer_Cloned_1              GCTTTACTAGTAGTCTCTTGGGAGGAGCTTGCGGGACCTAGCTCCACCGT
AtSHN1_NosTer_Cloned_2_             GCTTTACTAGTAGTCTCTTGGGAGGAGCTTGCGGGACCTAGCTCCACCGT
AtSHN1_NosTer_Vector_Control        GCTTTACTAGTAGTCTCTTGGGAGGAGCTTGCGGGACCTAGCTCCACCGT
                                    **************************************************

AtSHN1_NosTer_Cloned_1              CATGACCCAGCTGGAGTCAGACTCTGAACCGGCCCGTTTCTGCCAGACGC
AtSHN1_NosTer_Cloned_2_             CATGACCCAGCTGGAGTCAGACTTTGAACCGGCCCGTTTCTGCCAGACGC
AtSHN1_NosTer_Vector_Control        CATGACCCAGCTGGAGTCAGACTTTGAACCGGCCCGTTTCTGCCAGACGC
```

```
                                  *********************** **************************

AtSHN1_NosTer_Cloned_1            CGATATGGGAGCTGGCTGTGTCAAGACGGAGGCAGGTGAGGGATGGGGAA
AtSHN1_NosTer_Cloned_2_           CGATATGGGAGCTGGCTGTGTCAAGACGGAGGCAGGTGAGGGATGGGGAA
AtSHN1_NosTer_Vector_Control      CGATATGGGAGCTGGCTGTGTCAAGACGGAGGCAGGTGAGGGATGGGGAA
                                  **************************************************

AtSHN1_NosTer_Cloned_1            GGAGACTTGCAGCATTTCCTCAGTTTGGCGCTGAGGATGGAAGAGAGCGA
AtSHN1_NosTer_Cloned_2_           GGAGACTTGCAGCATTTCCTCAGTTTGGCGCTGAGGATGGAAGAGAGCGA
AtSHN1_NosTer_Vector_Control      GGAGACTTGCAGCATTTCCTCAGTTTGGCGCTGAGGATGGAAGAGAGCGA
                                  **************************************************

AtSHN1_NosTer_Cloned_1            TGAAGATGATGTTGAGGATGACATTGTGGACGAAGCTGAAA-TATCGGTT
AtSHN1_NosTer_Cloned_2_           TGAAGATGATGTTGAGGATGACATTGTGGACGAAGCTGAAA-TATCGGTT
AtSHN1_NosTer_Vector_Control      TGAAGATGATGTTGAGGATGACATTGTGGACGAAGCTGAAAATATCGGTT
                                  ***************************************** ********

AtSHN1_NosTer_Cloned_1            TTGCCCTCGGAAGTTTCTCC-GGTGTTGTTGTTGTTGAGGGGAAAGTTGG
AtSHN1_NosTer_Cloned_2_           TTGCCCTCGGAAGTTTCTCCCGGTGTTGTTGTTGTTGAGGGGAAAGTTGG
AtSHN1_NosTer_Vector_Control      TTGCCCTCGGAAGTTTCTCC-GGTGTTGTTGTTGTTGAGGGGAAAGTTGG
                                  ******************** *****************************

AtSHN1_NosTer_Cloned_1            TTTTGGCGTTGCGGCCGCTCATTAAAACGGCGGCCTCGTCGTATGCTCTT
AtSHN1_NosTer_Cloned_2_           TTTTGGCGTTGCGGCCGCTCATTAAAACGGCGGCCTCGTCGTATGCTCTT
AtSHN1_NosTer_Vector_Control      TTTTGGCGTTGCGGCCGCTCATTAAA-CGGCGGCCTCGTCGTATGCTCTT
                                  ************************** ***********************

AtSHN1_NosTer_Cloned_1            TGAATATATATATATATATAGTAGAATTTTTAAGCAATAACTTATTGATA
AtSHN1_NosTer_Cloned_2_           TGAATATATATATATATA--GTAGAATTTTTAAGCAATAACTTATTGATA
AtSHN1_NosTer_Vector_Control      TGAATATATATATATATATAGTAGAATTTTTAAGCAATAACTTATTGATA
                                  ******************  ******************************

AtSHN1_NosTer_Cloned_1            TTTACGTATCTAGATGTTAAAATGATTTTATGTGTTAGCTAAGAAGAGGA
AtSHN1_NosTer_Cloned_2_           TTTACGTATCTAGATGTTAAAATGATTTTATGTGTTAGCTAAGAAGAGGA
AtSHN1_NosTer_Vector_Control      TTTACGTATCTAGATGTTAAAATGATTTTATGTGTTAGCTAAGAAGAGGA
                                  **************************************************

AtSHN1_NosTer_Cloned_1            AAACAACAAAGGAAGATAAACCAGAAATGAGTTAGCTACGATCATGTATA
AtSHN1_NosTer_Cloned_2_           AAACAACAAAGGAAGATAAACCAGAAATGAGTTAGCTACGATCATGTATA
AtSHN1_NosTer_Vector_Control      AAACAACAAAGGAAGATAAACCAGAAATGAGTTAGCTACGATCATGTATA
                                  **************************************************

AtSHN1_NosTer_Cloned_1            TATATGTATGTGTGGTTGTGTACTTTAATGGATCAGAGAGAGGAAATAAA
AtSHN1_NosTer_Cloned_2_           TATATGTATGTGTGGTTGTGTACTTTAATGGATCAGAGAGAGGAAATAAA
AtSHN1_NosTer_Vector_Control      TATATGTATGTGTGGTTGTGTACTTTAATGGATCAGAGAGAGGAAATAAA
                                  **************************************************

AtSHN1_NosTer_Cloned_1            TGAACAACGCAGAGAAAAAGGAAAGAAAGAAGAAAGGTACAAGAGAGGAT
AtSHN1_NosTer_Cloned_2_           TGAACAACGCAGAGAAAAAGGAAAGAAAGAAGAAAGGTACAAGAGAGGAT
AtSHN1_NosTer_Vector_Control      TGAACAACGCAGAGAAAAAGGAAAGAAAGAAGAAAGGTACAAGAGAGGAT
                                  **************************************************

AtSHN1_NosTer_Cloned_1            GACGAATCTCAGCGACCCAAGAACCCCAATGGCGTTGCCTGACACCTCTG
AtSHN1_NosTer_Cloned_2_           GACGAATCTCAGCGACCCAAGAACCCCAATGGCGTTGCCTGACACCTCTG
AtSHN1_NosTer_Vector_Control      GACGAATCTCAGCGACCCAAGAACCCCAATGGCGTTGCCTGACACCTCTG
                                  **************************************************

AtSHN1_NosTer_Cloned_1            AACTTCTTCGTCTGTACCATTCTTACTTACTCTGTG
AtSHN1_NosTer_Cloned_2_           AACTTCTTCGTCTGTACCATTCTTACTTACTCTG--
AtSHN1_NosTer_Vector_Control      AACTTCTTCGTCTGTACCATTCTTACTTACTCTGTG
                                  **********************************
```
